# Training received, knowledge, and use of Silver Diamine Fluoride among Italian dentists: a nationwide survey

**DOI:** 10.1186/s12903-024-05181-x

**Published:** 2025-01-20

**Authors:** Claudia Salerno, Giulio Conti, Silvia Cirio, Cinzia Maspero, Andrea Senna, Guglielmo Campus, Maria Grazia Cagetti

**Affiliations:** 1 Department of Restorative, Preventive and Pediatric Dentistry, University of Bern, Freiburgstrasse 7, Bern, 3012 Switzerland; 2 Department of Biomedical, Surgical and Dental Sciences, University of Milan, Milan, Italy; 3 Graduate School for Health Sciences, University of Bern, Bern, Switzerland; 4 Department of Medicine and Surgery, University of Insubria, Varese, 21100 Italy; 5 Fondazione IRCCS Ca‘ Granda, Ospedale Maggiore Policlinico, Milan, Italy; 6 Italian National Commission of the Dental Board, Milan, Italy; 7 Department of Cariology, Saveetha Dental College and Hospitals, SIMATS, Poonamallee High Road, Chennai, 600077 India; 8 Department of Cariology, Institute of Odontology at Sahlgrenska Academin, University of Gothemburg, Gothenburg, Sweden

**Keywords:** Pediatric dentistry, Silver diamine fluoride, Dental caries, Dental education

## Abstract

**Objective:**

To investigate the education, knowledge and behaviour of Italian dentists regarding Silver Diamine Fluoride (SDF).

**Methods:**

A cross-sectional study was conducted from January to December 2022, through an online survey linked to an online continuing medical education (CME) course sent to Italian dentists. A priori power analysis estimated the necessary sample to be 1480 dentists with an anticipated frequency of 50% and a power of 99.99%. The questionnaire included 46 questions on participants ‘ demographic characteristics, training received, clinical knowledge of SDF, and attitudes and behaviours regarding its use. Descriptive statistics, bivariate, and mutlivariable regression analyses were performed to determine the association between the variables.

**Results:**

The response rate was 6.1% with 3876 respondents, evenly distributed geographically. Less than 10% of respondents had received training at undergraduate, postgraduate or masters level. A minority of dentists were familiar with the use of SDF for the treatment of dentine hypersensitivity (19.0%) and for the treatment of caries in children (22.2%) and adults (15.7%). The percentage of dentists who reported SDF use at least once was 20.6%. On mutlivariable analysis (χ^2^_(11)_ = 995.9 *p-*value < 0.01), dentists who used SDF were positively associated with those who cared for patients with special needs, those who received good undergraduate or postgraduate training, and those who knew how to use SDF (*p* < 0.01). A second mutlivariable analysis (χ^2^_(11)_ = 47.9 *p-*value < 0.01) revealed that younger respondents were associated with good training and knowledge of the use of SDF received during undergraduate studies, while older respondents were associated with good training received on managing hypersensitivity and caries in adults (*p* < 0.01).

**Conclusions:**

Overall, Italian dentists ‘ education, knowledge, and use of SDF were relatively poor. The majority of the sample ‘s responses were not consistent with scientific evidence. The use of SDF among Italian dentists is still far from being a reality. In Italy, it is necessary to increase training on SDF, primarily through the university, to hopefully increase its use, especially in non-invasive caries treatment.

**Supplementary Information:**

The online version contains supplementary material available at 10.1186/s12903-024-05181-x.

## Introduction

The definition of dental caries as a manageable chronic disease broadens the concept of primary caries prevention that is based on sugar reduction and proper daily brushing with fluoridated toothpaste [1]. Caries should be treated by a medical rather than a surgical approach, although surgery is still the most common caries management procedure [2, 3]. While a surgical approach restores cavities, a medical approach handles caries disease. Dental caries is managed medically by evaluating the risk of cavities, detecting them early, classifying lesions as active or inactive, and offering advice on food, oral hygiene, and products to stop active lesions [4].

Silver Diamine Fluoride (SDF) appears to be a potential medical approach because it acts on the microbiome as well as the hard tissues; SDF promotes remineralisation of enamel and dentin, reduces the number of bacteria in the biofilm, and hinders the degradation of collagen, thereby halting the caries progression [5, 6]. In 2014, the Food and Drug Administration classified SDF as a Class II medical device and approved its use for the treatment of tooth sensitivity; three years later, the American Academy of Paediatric Dentistry (AAPD) endorsed its use to stop cavitated lesions in primary teeth [7, 8]. The World Health Organization has included SDF together with fluoride toothpaste and glass ionomer cement in its list of essential medicines. SDF is considered a minimally invasive, inexpensive, simple, and effective technique that can help reduce stress and anxiety, especially in young children [9]. In addition, SDF is recommended for the treatment of caries in patients with special care needs and for the arrest of root carious lesions in elderly patients, particularly those who are bedridden or living in elderly community centres [10, 11].

SDF at a concentration of 38% applied to carious lesions in primary teeth has proven to be as effective as glass-ionomer cement restorations and more effective in preventing dental caries than no treatment or fluoride varnish [12, 13]. In addition, it is easy to use in a community setting; this aspect is of great importance as dental caries is still one of the most common non-communicable diseases worldwide [14]. High rates of untreated caries lesions in children are recorded in Italy, particularly in those with non-European backgrounds [15, 16].

Depending on the area (region), the Italian National Health Service covers a limited range of dental procedures. While some regions provide free dental care for children up to the age of 14, in the majority of regions, dental care in public facilities is only available for people of any age having a medical condition or with financial disadvantages. Consequently, about 95% of dental care is provided by private dentists, and this expenditure, together with pharmaceuticals, accounts for the majority of private health expenditure. As a result, 5.7% of people do not seek dental treatment because of financial constraints [12]. This situation calls for inexpensive but effective caries treatments such as SDF. Only one product containing 38% SDF is available on the Italian market and it is only recommended for the treatment of hypersensitivity of teeth. By 2016, almost all US paediatric dentistry residency programmes had begun to include SDF-related content in their curricula, with a quarter of programmes using it in the clinical setting [17]. The European Core Curriculum in Cariology states that upon graduation, a dentist should be able to select the appropriate treatment option based on a sound knowledge of non-surgical and surgical treatment options. However, no specific reference was made to SDF [18]. The Italian Core Curriculum in Cariology was created in 2018 by an Italian expert panel, which kept the content of the European document the same, but modified its form for the Italian setting. [19].

Studies investigating dentists ‘ training, knowledge and use of SDF by dentists are scarce and most of them have been conducted among paediatric dentists [19, 20]. The actual use of SDF among dentists, especially those in private practice, remains unclear [21].

Based on these premises, the aim of this survey was to investigate the training experience, knowledge, attitudes and use of SDF by Italian dentists. The survey will make it possible to assess whether increased use corresponds to increased training and knowledge in this area.

## Materials and methods

### Study design and sample

The study was designed as an observational, questionnaire-based, cross-sectional study; it complied with the Declaration of Helsinki and was conducted after approval by the Ethics Committee (Ethics Committee Board of the University of Sassari, Sassari, Italy, N°AOU_SS 94 of 11 November 2021).

The questionnaire was developed in Italian using two validated questionnaires [19, 22]. The validated questionnaires were forward translated into Italian by two native translators; then, a consensus version was identified and translated back into English by an independent person not involved in the study. A quantitative analysis of the accuracy of the questionnaire was carried out by submitting it to 12 experts (5 dentists specialised in paediatric dentistry with more than 5 years of experience, 4 academics and 3 clinical researchers). The quantitative content validity of each item was assessed using the content validity index (CVI) and the content validity ratio (CVR) [23]. Finally, the Scale Content Validity Index (S-CVI) was calculated using the universal agreement method. Based on experts ‘ opinions, the S-CVI and S-CVR for the entire tool were 1.00 and 0.98, respectively. The questionnaire was pre-tested on a small sample of 15 general dentists who were not included in the survey. After completing the questionnaire, they were contacted to find out if they had experienced any difficulty in understanding the questions and were asked to give a comprehension score from 1 (extreme difficulty) to 5 (no difficulty), with a result of 4.4 ± 0.3. The intraclass correlation coefficient (ICC) was calculated among 15 dentists (not included in the sample) after 60 days to assess test–retest validity.

The new questionnaire comprised 46 questions divided into four domains (English version questionnaire, Supplementary material). The first domain consisted of 6 questions regarding the respondent ‘s demographic characteristics. The second one consisted of 10 questions investigating the received training and clinical knowledge on SDF, such as the quality of their training during undergraduate, postgraduate, and professional post-degree courses. The third section covered dentists ‘ attitudes on SDF through 16 questions. The final section, consisting of 14 questions, assessed the dentists ‘ behaviour regarding SDF use in clinical practice. The possible answers were dichotomous (yes/no) or on a Likert scale (four options). Sentinel questions were introduced to assess the consistency and diligence of the respondents in filling out the questionnaire. The answers to these questions had not to be contradictory (*e.g.*, who answered “never “ to the question “How often have you used SDF for…? “ had to state “I don ‘t use SDF? “ to the question “What kind of SDF protocol do you use? “).

Since all licensed dentists in Italy must have an e-mail account, all of them were contacted using their e-mail addresses obtained from the registers of the Italian Federation of Doctors and Dentists. The e-mail contained an invitation to participate in an online continuing medical education (CME) course to which the questionnaire was linked. According to Italian law, the following categories are registered as dentists: graduates in Medicine with a specialisation in Dentistry and enrolled in Medicine before 1980; graduates in Medicine between 1980 and 1985; graduates in Dentistry from 1985 to the present. No exclusion criteria were applied except for non-acceptance of informed consent.

Non-respondents received no further invitations or follow-ups. Before the first question, the study ‘s goal was explained, and according to Italian data protection law, dentists were required to sign an online informed consent form. If they declined to sign the consent, the survey was immediately closed. The survey was conducted from January 2022 to December 2022 [22]. An a priori power analysis was used to calculate the sample size using OpenEpi. Given the national population of dentists of 63,883 [22], the minimum sample size resulted in 1480 dentists with an anticipated frequency of 50%, a power of 99.99%, a design effect of 1, and an alfa error of 0.05.

### Data analysis

All data collected were downloaded and imported into a Microsoft Excel® spreadsheet (Microsoft® Office 2016, Microsoft® Corp, Redmond, WA, USA) and quality checked to ensure accuracy. Descriptive statistics were calculated for all items to provide an overview of the results.

The response to the question on SDF use allowed respondents to be divided into two categories: those who used SDF and those who did not. These two categories were compared for all variables using the χ^2^ or Fisher exact tests. In bivariate and mutlivariable analyses, the answers ‘I don ‘t know ‘ or ‘not applicable ‘ were not considered, and answers were combined into two nominal categories as follows: age (≤ 40 years of age and > 40 years of age); type of patient prevalently treated (children or adults/elderly); Likert responses (very good/good vs little/not at all; strongly agree/agree *vs* disagree/strongly disagree; very often/often *vs* rarely/never). Cross-tabulations were calculated for items in the first and second domains of the questionnaire by bivariate analysis (independent variables). Considering the possibility of an association between knowledge acquired during undergraduate education and knowledge acquired during postgraduate education on SDF, dummy variables were created as the sum of the data (‘undergraduate education ‘and ‘postgraduate education ‘).

Mutlivariable predictive regression models (STATA ‘s probit command) were run using the forward procedure to assess the relationship between SDF use (dependent variable) and responses to other items as independent variables, and the relationship between respondents ‘age (dependent variable) and source of SDF knowledge (independent variables). The data were checked for multicollinearity using the Belsley-Kuh-Welsch technique. Heteroskedasticity and normality of the residuals were assessed using the White test and the Shapiro–Wilk test, respectively. The interaction model (likelihood ratio test statistic) assessed potential effect modifiers. A *p-*value < 0.05 was considered statistically significant.

## Results

A total of 3876 dentists completed the questionnaire, giving an overall response rate of 6.1%. Questionnaires with inconsistent responses to sentinel questions were excluded, and 3337 questionnaires were analysed, exceeding the number required for the power analysis (*n* = 1480).

**Fig. 1:**
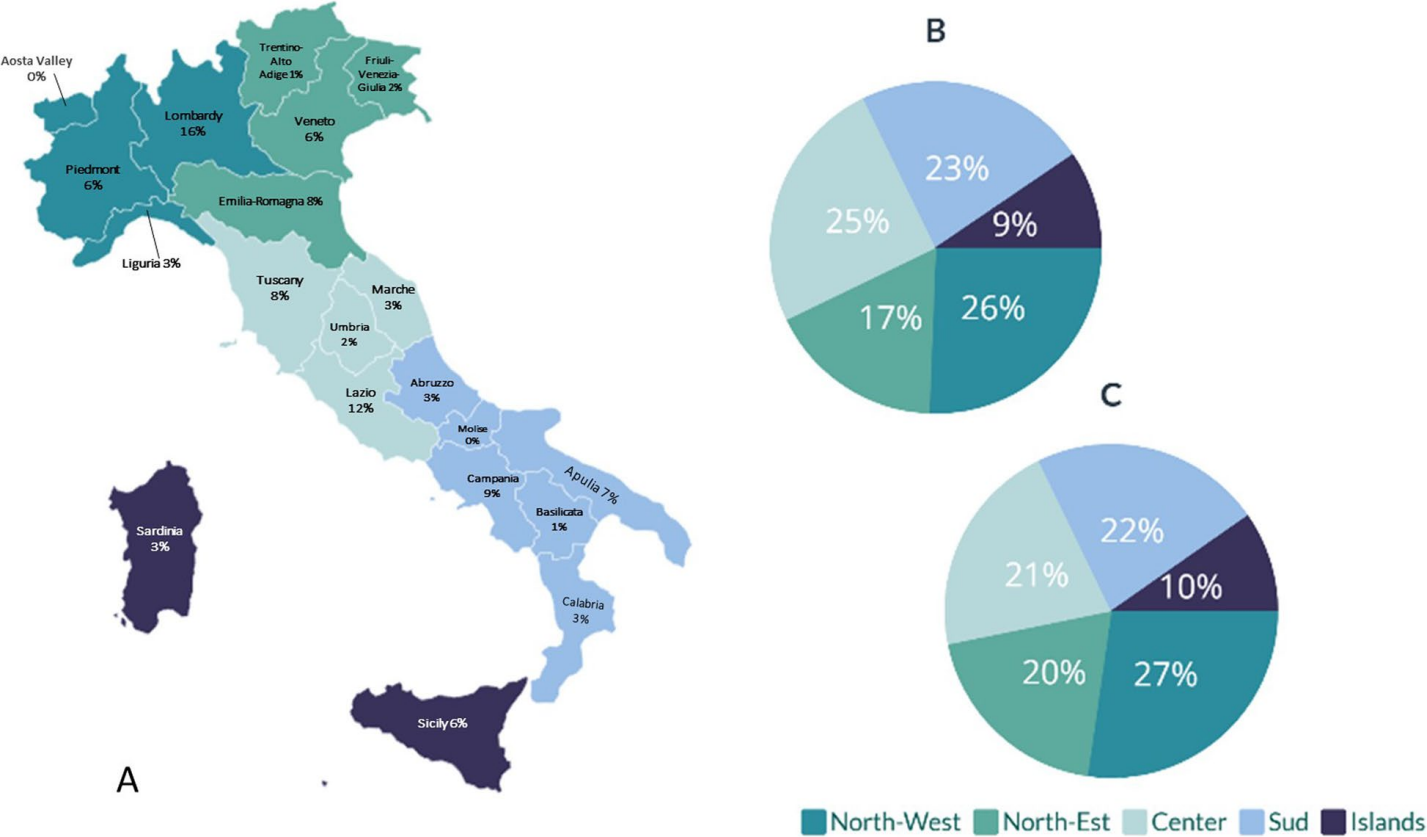
Distribution (%) of the sample in each region (**A**) and each macro-area (**B**); distribution (%) of dentists working in each macro-area reported by ISTAT (**C**)

The distribution of respondents across Italy (Fig. 1A, B) was homogeneous and proportional to the number of dentists per macro-area (Fig. 1C). Respondents were broadly representative of Italian dentists. Most (69.2%) were over 40 years of age and more than half (50.8%) had worked for more than 20 years. Most worked in private practice (86.9%) and in urban areas (67.9%). Almost all (82.6%) cared mainly for adults, and less than half (42.2%) cared for patients with special health care needs (Table S1, Supplementary material).

Table 1 shows the results for the 2nd, 3rd and 4th domains. Overall, respondents received little training in the use of SDF and their level of knowledge was low. Combining the Strongly Agree and Agree response categories, more dentists considered SDF to be an appropriate treatment option for the management of enamel cavitated lesions (61.6%) than for dentin lesions (35.3%). Many (25.5%) disagreed that SDF could be used without restorative treatment, and almost a third answered ‘I don ‘t know ‘. More respondents considered SDF a good treatment (yes/no) for posterior primary teeth (40.6%) than those who considered it a good treatment for anterior teeth (11.5%). The majority agreed that SDF is a suitable treatment for patients with behavioural problems (60.3%). Permanent discolouration and failure to restore dental anatomy were the main concerns of the respondents. Among respondents, 20.6% (*n* = 688) had used SDF; 73.7% reported they expected to increase its use. Fewer dentists used SDF often or very often.

**Table 1:** Participants expressed as number and percentage of the dentists’ education, attitudes toward SDF and behavior on its use in clinical practice (participants 3337)

Item	N (%)				
**Domain 2: training received and clinical knowledge on SDF**					
**How well were you educated about SDF during undergraduate course…**	Very well	Well	A little	Not at all	
in classroom settings?	29 (1.0)	203 (6.1)	768 (23.0)	2337 (70.0)	
in clinical settings?	32 (1.0)	188 (5.6)	668 (20.0)	2449 (73.4)	
**How well were you educated about SDF after graduation…**	Very well	Well	A little	Not at all	
in Continuing Education Courses?	68 (2.0)	280 (8.4)	1022 (30.6)	1967 (58.9)	
with dental journals/other publications?	84 (2.5)	396 (11.9)	1392 (41.7)	1465 (43.9)	
through dental organizations?	69 (2.1)	355 (10.6)	1213 (36.4)	1700 (50.9)	
with online resources?	118 (3.5)	485 (14.5)	1303 (39.1)	1431 (42.9)	
in post-graduate courses?	73 (2.2)	222 (6.6)	799 (23.9)	2243 (67.2)	
**How much do you know about how SDF is used…**	Very well	Well	A little	Not at all	
for treatment of tooth hypersensitivity?	66 (2.0)	570 (17.1)	1496 (44.8)	1205 (36.1)	
to treat dental caries in pediatric patients?	101 (3.0)	639 (19.1)	1508 (45.2)	1089 (32.6)	
to treat dental caries in adult patients?	66 (2.0)	457 (13.7)	1504 (45.1)	1310 (39.3)	
**Domain 3: dentist’s attitudes toward SDF**					
**How much do you disagree/agree with the following statements? SDF can be used…**	Strongly agree	Agree	Disagree	Strongly disagree	I don’t know
to arrest non-cavitated lesions	416 (12.5)	1693 (50.7)	346 (10.4)	85 (2.5)	797 (23.9)
to arrest enamel cavitated lesion	332 (10.0)	1723 (51.6)	401 (12.0)	51 (1.5)	830 (24.9)
to arrest dentin cavitated lesion	194 (5.8)	1159 (34.7)	899 (26.9)	164 (4.9)	921 (27.6)
to arrest cavitated root caries	170 (5.1)	1061 (31.8)	858 (25.7)	254 (7.6)	994 (29.8)
after removing infected soft dentin	180 (5.4)	1224 (36.7)	822 (24.6)	161 (4.8)	950 (28.5)
without restorative treatment	152 (4.6)	1259 (37.7)	728 (21.8)	123 (3.7)	1075 (32.2)
**Is SDF a good treatment for lesions that are…**	Yes	No	I don’t know		
in the aesthetic zone on primary teeth?	385 (11.5)	2005 (60.1)	947 (28.4)		
in the posterior zone on primary teeth?	1353 (40.5)	1037 (31.1)	947 (28.4)		
in the aesthetic zone on permanent teeth?	310 (9.3)	2080 (62.3)	947 (28.4)		
in the posterior zone on permanent teeth?	1648 (49.4)	742 (22.2)	947 (28.4)		
**Is SDF a good alternative treatment for patient…**	Yes	No	I don’t know		
special needs?	1517 (45.4)	963 (28.9)	857 (25.7)		
with severe dental anxiety?	1409 (42.2)	1071 (32.1)	857 (25.7)		
with behavioral issues?	2012 (60.3)	468 (14.0)	857 (25.7)		
low-income?	788 (23.6)	1692 (50.7)	857 (25.7)		
taking bisphosphonate?	342 (10.2)	2138 (64.1)	857 (25.7)		
during/shortly after radiotherapy or chemotherapy?	556 (16.6)	1924 (57.7)	857 (25.7)		
**Domain 4: dentists’ behavior on the use of SDF in clinical practice**					
**What are your doubts about SDF?**	Yes	No	I don’t know		
Poor scientific evidence	217 (6.5)	2377 (71.2)	743 (22.3)		
Permanent discoloration of treated teeth	2228 (66.7)	306 (11.0)	743 (22.3)		
Cost for the patients	134 (4.0)	2460 (73.7)	743 (22.3)		
Failure to restore functional anatomy of teeth	1100 (32.9)	1494 (44.8)	743 (22.3)		
Concern for patient satisfaction	860 (25.7)	1734 (52.0)	743 (22.3)		
Off-label use	292 (8.7)	2302 (69.0)	743 (22.3)		
**Do you use SDF?**	Yes	No			
	688 (20.62)	2649 (79.38)			
**How often did you use SDF…**	Very often	Often	Rarely	Never	
to treat tooth sensitivity?	14 (0.4)	158 (4.7)	455 (13.6)	2710 (81.2)	
to prevent dental caries?	14 (0.4)	179 (5.4)	414 (12.4)	2730 (81.8)	
to arrest dental caries in primary teeth?	21 (0.6)	207 (6.2)	412 (12.4)	2697 (80.8)	
to arrest dental caries in permanent teeth?	13 (0.4)	148 (4.4)	406 (12.7)	2770 (83.0)	
to treat cavitated caries lesion without restoration?	33 (1.0)	156 (4.7)	348 (10.4)	2800 (83.9)	
**What kind of SDF’s protocol do you use?**					
One-shot	144 (4.3)				
An application every week for 1 month	23 (0.7)				
An application every 3 months	210 (6.3)				
An application every 6 months	71 (2.1)				
An application every 12 months	24 (0.7)				
Based on lesion characteristics/patient ‘s caries risk	216 (6.5)				
I don ‘t use SDF	2649 (79.4)				
**Do you expect to increase your future usage of SDF?**	Yes	No			
	2461 (73.7)	876 (26.3)			

A cross-sectional tabulation was performed using SDF (yes or no) and the variables of domains 1 and 2 to assess whether greater use corresponded to greater training and knowledge in this area (Table 2). Almost all variables in domains 3 and 4 were statistically different between those who used SDF and those who did not; the results of the bivariate analysis according to SDF use for domains 3 and 4 within the two groups are shown in Table S2 (Supplementary material).

**Table 2:** Cross tabulation of the distribution of the participants according to SDF use of 1st and 2nd domain of the questionnaire. The association was evaluated by *χ*^2^ test

	No SDF use	SDF use	Total
	n (%)	n (%)	n (%)
**Age**			
≤ 40 years of age	850 (82.9)	176 (17.1)	1026 (100)
> 40 years of age	1799 (77.9)	512 (22.1)	2311 (100)
* χ*^*2*^_*(1)*_ = *10.86 p* < *0.01*			
**Work experience**			
< 5 years	350 (85.6)	59 (14.4)	409 (100)
5–10 years	342 (78.1)	96 (21.9)	438 (100)
11–20 years	655 (82.6)	138 (17.4)	793 (100)
> 20 years	1302 (76.7)	395 (23.3)	1697 (100)
* χ*^*2*^_*(3)*_ = *22.37 p* < *0.01*			
**Practice/employment situation**			
Private clinic	2323 (80.1)	577 (19.9)	2900 (100)
Public clinic	112 (70.0)	48 (30.0)	160 (100)
Both	214 (77.3)	63 (22.7)	277 (100)
* χ*^*2*^_*(2)*_ = *10.29 p* < *0.01*			
**Workplace**			
Big city	545 (81.2)	126 (18.8)	671 (100)
Small/ moderate city	1269 (79.6)	326 (20.4)	1595 (100)
Rural	835 (78.0)	236 (22.0)	1071 (100)
* χ*^*2*^_*(2)*_ = *2.73 p* = *0.25*			
**Type of patients prevalently treated**			
Children	363 (77.9)	103 (22.1)	466 (100)
Adults and elderly	2286 (79.6)	585 (20.4)	2871 (100)
* χ*^*2*^_*(1)*_ = *0.73 p* = *0.39*			
**Treating special needs patients**			
No	1634 (84.7)	295 (15.3)	1929 (100)
Yes	1015 (72.1)	393 (27.9)	1408 (100)
* χ*^*2*^_*(1)*_ = *79.19 p* < *0.01*			
**Undergraduate education**			
A little/ Not at all	2577 (84.2)	485 (15.8)	3062 (100)
Well/ Very well	71 (26.2)	203 (73.8)	275 (100)
* χ*^*2*^_*(1)*_ = *518.28 p* < *0.01*			
**Postgraduate education**			
A little/ Not at all	2298 (89.8)	261 (10.2)	2559 (100)
Well/ Very well	351 (45.1)	427 (54.9)	778 (100)
* χ*^*2*^_*(1)*_ = *727.88 p* < *0.01*			
**Knowledge on SDF use for treatment of tooth hypersensitivity**			
A little/ Not at all	2384 (88.3)	317 (11.7)	2701 (100)
Well/ Very well	265 (41.7)	371 (58.3)	636 (100)
* χ*^*2*^_*(1)*_ = *682.94 p* < *0.01*			
**Knowledge on SDF use for treatment of caries in pediatric patients**			
A little/ Not at all	2322 (89.4)	275 (10.6)	2597 (100)
Well/ Very well	327 (44.2)	413 (55.8)	740 (100)
* χ*^*2*^_*(1)*_ = *719.59 p* < *0.01*			
**Knowledge on SDF use for treatment of caries in adult patients**			
A little/ Not at all	2468 (87.7)	346 (12.3)	2814 (100)
Well/ Very well	181 (34.6)	342 (65.4)	523 (100)
* χ*^*2*^_*(1)*_ = *759.70 p* < *0.01*			

Table 3 shows how dentists who reported using SDF were positively associated with those who cared for patients with special needs (*p* < 0.01), those who received good training during the undergraduate course or postgraduate courses and those who had good knowledge about the use of SDF for tooth sensitivity, caries in children and adults (*p* < 0.01). A second analysis was performed to investigate whether the age of the participants might play a role in the sources of information due to the relatively recent introduction of SDF in Italy. Younger respondents were more likely to have received training before graduation (*p* < 0.01) and to report knowing how to use SDF for caries treatment in children (*p* = 0.01). Older respondents were more likely to report knowing how to use SDF for the treatment of tooth sensitivity (*p* = 0.01) and caries in adults (*p* < 0.01) (Table 3).

**Table 3:** Coefficient estimates of the model (forward predictive regression models) for use of SDF and the age of the participants

Covariates	Coefficient	SE	_95%_CI	*p-*value
**Use of SDF**				
** Age range < 40**	0.1	0.1	[-0.1; 0.3]	0.3
** Work experience**	0.1	0.1	[-0.0; 0.2]	0.2
** Practice/employment situation**	0.0	0.0	[-0.1; 0.1]	0.9
** Workplace**	0.0	0.0	[-0.7; 0.1]	0.8
** Type of patient prevalently treated**	< 0.01	0.1	[-0.2; 0.2]	1.0
** Treating Special needs patients**	0.2	0.1	[0.1; 0.3]	< 0.01
** Undergraduate education**	0.8	0.1	[0.6; 1.0]	< 0.01
** Postgraduate education**	0.7	0.1	[0.6; 0.9]	< 0.01
** Knowledge on SDF use for treatment of tooth hypersensitivity**	0.3	0.1	[0.1; 0.5]	< 0.01
** Knowledge on SDF for treatment of caries in pediatric patients**	0.5	0.1	[0.3; 0.6]	< 0.01
** Knowledge on SDF use for treatment of caries in adult patients**	0.4	0.1	[0.2; 0.6]	< 0.01
* constant*	-1.9	0.2	[-2.4; -1.5]	< 0.01
* Log likelihood* = *-1200.1; N of observation* = *3337; χ*^*2*^_*(11)*_ = *995.9 p-value* < *0.01; Pseudo R*^*2*^ = *0.3*				
**Age of participants**				
** Undergraduate education**	-0.6	0.1	[-0.8; -0.4]	< 0.01
** Postgraduate education**	0.1	0.1	[-0.0; 0.3]	0.1
** Knowledge on SDF for hypersensitivity**	0.3	0.1	[0.1; 0.5]	< 0.01
** Knowledge on SDF for caries in children**	-0.2	0.1	[-0.4; -0.1]	0.0
** Knowledge on SDF for caries in adults**	0.3	0.1	[0.1; 0.5]	0.0
* constant*	0.5	0.0	[0.4; 0.5]	< 0.01
*Log likelihood = -2035.2; N of observation = 3337; χ2(11) = 47.9 p-value < 0.01; Pseudo R2 = 0.0*				

## Discussion

Overall, Italian dentists ‘ knowledge of SDF was relatively low. The majority of the sample ‘s responses were inconsistent with the scientific evidence. This finding suggests that although part of the sample claims to be aware of the use of SDF, the Italian dental population has difficulty believing in the effectiveness of medical caries treatment for more advanced lesions, such as cavitated dentin lesions.

Regarding education on SDF, almost all respondents reported that they had not received any education on SDF in lectures and clinical practice during their undergraduate studies. Similar results have been reported in a survey in Saudi Arabia, while worse results have been reported in the USA [19, 24]. This finding is not surprising, as SDF was licensed in 2014, and less than a quarter of the respondents have been practising for less than five years, so it can be assumed that they were trained in dental school after 2015 [8].

The increase in training experience in SDF in paediatric dentistry residency programmes compared to undergraduate programmes is not surprising, as one of the main target groups for SDF is children. Training on SDF in paediatric dentistry residency programmes is expected to increase soon, as the AAPD approved its use for caries treatment in 2017 [7]. However, after graduation, in the present sample, most of the knowledge was acquired individually through dental journals or other publications. In the USA, on the other hand, information was more likely to be passed on through continuing education courses and dental organisations [19]. Overall, Italian dentists ‘ knowledge of SDF was very low, in line with a similar study of general dentists [22].

Despite the relatively low level of training on SDF, respondents ‘ self-reported knowledge of its use was slightly higher than expected. However, although more than a quarter of respondents claimed to know what SDF is used for in dentistry, the majority of the sample gave answers that were inconsistent with the scientific evidence. The survey results suggest that although a proportion of the sample claims to be aware of the use of SDF, the general dental population has difficulty believing in the efficacy of medical caries therapy and non-invasive treatment of more severe lesions. This finding differs from what was found among American dentists, where the vast majority considered SDF to be an effective treatment for arresting both enamel and dentin lesions. However, this result is not surprising as the survey participants were members of the American Academy of Paediatric Dentistry [19].

In the present study, as in other surveys, there was a high level of agreement with the statements that SDF is suitable for the treatment of carious lesions in patients with behavioural problems, dental phobia and patients with special needs [17, 19].

One-fifth of the dentists reported using SDF at least once, while a negligible percentage reported using it frequently in all the proposed scenarios. The use of SDF was more preferred by American dentists than by Italian dentists, although the frequency of use varied [17, 19].

Most dentists reported using SDF according to the clinical situation, once a year or every 3 months, reflecting the different proposed application protocols. A recent systematic review compared repeated application of SDF with a single application at least once every six months and concluded that repeated application increased its efficacy [12]. Despite the lack of training received during and after graduation, the low level of knowledge and the doubts expressed by dentists about the use of SDF, more than two-thirds of dentists indicated a willingness to increase the use of SDF in clinical practice. This result differs from that found in Saudi Arabia, where the reduced use of SDF is likely to persist, as dentists do not seem motivated to increase its use in the future [25]. This result can be partly explained by the fact that respondents saw the discolouration of teeth caused by SDF as a deterrent to its use.

The use of SDF was higher among dentists who had received adequate training and among young dentists who had received more training during their undergraduate studies. As previously reported, areas of educational experience and teaching practices can provide useful information in identifying the source of gaps in dentists ‘current knowledge [19, 22]. These data support the hypothesis that dentists have realised the vast potential of this solution and support the need to build and increase appropriate training on its use.

Dentists who treat individuals with special needs reported greater use of SDF than those who do not routinely treat these patients. SDF is an alternative treatment for dental caries if other approaches are not available; because of this, it is suitable for treating children with fear and anxiety and uncooperative patients of any age [12].

Except for patients who have received or are receiving radiation therapy, where no significant differences were found, the results of this national survey demonstrated, as predicted, that dentists who use SDF have better knowledge of the indications for its correct use, the type of lesions that can be treated, the location of the lesions, and the type of patients in whom it is most recommended.

Dentists who do not use SDF consider patient or parent satisfaction, failure to restore tooth anatomy, and tooth discolouration as their main concerns. These findings are consistent with similar studies [7, 25]. The use of SDF for the treatment of caries in the Italian context is supported only at the scientific level and mainly in English. These aspects may limit the dissemination and use of the product for caries treatment. Scientific evidence from research typically takes an average of 17 years to be translated into clinical practice [26]. Although the use of SDF has been described since the 1960s, it has only recently attracted scientific and clinical interest in Europe and has only been available on the Italian market for the past five years. These aspects may suggest that the use of this product will increase in the coming years.

The large sample size strengthens the results of the present work and allows them to be generalised to the entire population of Italian dentists. The limitations of this questionnaire include the recent introduction of the product in Italy, which did not allow the necessary latency period for the use of the product to become widespread in the territory; the lack of data on the gender and social status of the participants, which could have contributed to an in-depth analysis of the reasons for the reduced knowledge and use of the product. Another possible limitation is the method of recruitment, which may have contributed to the low response rate. Not all Italian dentists received a formal invitation to participate in the survey, but only those willing to take the course were enrolled. In addition, a further explanation for the low response rate could be due to a lack of knowledge of the topic and, thus, low interest in filling out the questionnaire. Nonetheless, the sample size was large and equally distributed throughout Italy, mitigating any potential bias resulting from the relatively low response rate.

To increase the knowledge and use of SDF in Italy, universities need to update their cariology curriculum by including the teaching of SDF among non-invasive caries treatments in both undergraduate and postgraduate courses. In addition, introducing more SDF-containing products in the market could promote awareness of SDF through advertising by competing companies. From a financial perspective, neither the community nor dentists would incur any expenses as a result of the information dissemination tactics listed above. However, it would benefit patients, especially those undergoing treatment under general anaesthesia, given the low cost of this treatment strategy [27]. Further investigation is needed to monitor how Italian dentists ‘ education, knowledge, and attitudes regarding SDF will change.

## Conclusion

Overall, the survey results highlighted concerns about appropriate use, inadequate training during and after graduation, and lack of knowledge, so that non-invasive caries treatment with SDF is far from being a known and widely used option. Although several participants claimed to be aware of the use of SDF, the Italian dental community may find it difficult to accept the effectiveness of non-invasive treatment of more severe lesions. The results of this survey also highlight the need for increased education to dispel any remaining misconceptions and doubts about this product.

## Supplementary Information

Supplementary Material 1.

## Data Availability

The datasets generated and/or analysed during this study will be available upon reasonable request.
