# Supplementary material for: Training received, knowledge, and use of Silver Diamine Fluoride among Italian dentists: a nationwide survey
Source: BMC Oral Health. 2025 Jan 20;25:106. doi: 10.1186/s12903-024-05181-x (PMC11748836; doi:10.1186/s12903-024-05181-x)
Supplement: Supplementary file 1 — Supplementary Material 1. [file 12903_2024_5181_MOESM1_ESM.docx]

Supplementary materials

Is Silver Diamine Fluoride a realistic choice for Italian dentists? A National survey

Indices

|  | *Page* |
| --- | --- |
| English version questionnaire | 1 |
| Table S1: Demographic characteristics expressed as number of participants and percentage. (Participants 3337) | 5 |
| Table S2: Reported use of SDF (yes or no) by questionnaire items (Domains 3 and 4) | 6 |

English version questionnaire

|  | **Item** | **Answers** | | | | | | | | | | | | | | | | | | | | | | | | |
| --- | --- | --- | --- | --- | --- | --- | --- | --- | --- | --- | --- | --- | --- | --- | --- | --- | --- | --- | --- | --- | --- | --- | --- | --- | --- | --- |
| **Domain 1** | How old are you? | <30 years | | | 31-40 years | | | | | 41-50 years | | | | | | 51-60 years | | | | | > 60 years | | | | | |
|  | How many years have you been in practice? | < 5 years | | | | 5-10 years | | | | | | 11-20 years | | | | | | | > 20 years | | | | | | | |
|  | What is your practice/employment situation? | Private clinic | | | | | | | Public clinic | | | | | | | | Both | | | | | | | | | |
|  | What is your workplace? | Big city | | | | | | | Small/ Moderate city | | | | | | | | Rural | | | | | | | | | |
|  | Which type of patients do you treat mainly? | Adults | | | | | | | Children (<14 years) | | | | | | | | Elderly (>65 years) | | | | | | | | | |
|  | Do you also treat special needs patients? | Yes | No | | | | |  | | |  | | | |  | | |  | | | | |  | | | |
| **Domain 2**  **Section 2** | How well were you educated about SDF during undergraduate course in classroom settings? | Very well | | | | Well | | | | | | A little | | | | | | | Not at all | | | | | | | |
|  | How well were you educated about SDF during undergraduate course in clinical settings? | Very well | | | | Well | | | | | | A little | | | | | | | Not at all | | | | | | | |
|  | How well were you educated about SDF after graduation in Continuing Education Courses? | Very well | | | | Well | | | | | | A little | | | | | | | Not at all | | | | | | | |
|  | How well were you educated about SDF after graduation with dental journals/other publications? | Very well | | | | Well | | | | | | A little | | | | | | | Not at all | | | | | | | |
|  | How well were you educated about SDF after graduation through dental organizations? | Very well | | | | Well | | | | | | A little | | | | | | | Not at all | | | | | | | |
|  | How well were you educated about SDF after graduation with online resources? | Very well | | | | Well | | | | | | A little | | | | | | | Not at all | | | | | | | |
|  | How well were you educated about SDF after graduation in post-graduate courses? | Very well | | | | Well | | | | | | A little | | | | | | | Not at all | | | | | | | |
|  | How much do you know about how SDF is used for treatment of tooth hypersensitivity? | Very well | | | | Well | | | | | | A little | | | | | | | Not at all | | | | | | | |
|  | How much do you know about how SDF is used to treat dental caries in pediatric patients? | Very well | | | | Well | | | | | | A little | | | | | | | Not at all | | | | | | | |
|  | How much do you know about how SDF is used to treat dental caries in adult patients? | Very well | | | | Well | | | | | | A little | | | | | | | Not at all | | | | | | | |
| **Domain 3** | How much do you disagree/agree with the following statements?  SDF can be used to arrest non-cavitated lesions | Strongly agree | | | | | Agree | | | | | | | Disagree | | | | | | Strongly disagree | | | | | I don’t know | |
|  | How much do you disagree/agree with the following statements? SDF can be used to arrest enamel cavitated lesions | Strongly agree | | | | | Agree | | | | | | | Disagree | | | | | | Strongly disagree | | | | | I don’t know | |
|  | How much do you disagree/agree with the following statements? SDF can be used to arrest dentin cavitated lesions | Strongly agree | | | | | Agree | | | | | | | Disagree | | | | | | Strongly disagree | | | | | I don’t know | |
|  | How much do you disagree/agree with the following statements? SDF can be used to arrest cavitated root caries | Strongly agree | | | | | Agree | | | | | | | Disagree | | | | | | Strongly disagree | | | | | I don’t know | |
|  | How much do you disagree/agree with the following statements? SDF can be used after removing infected soft dentin | Strongly agree | | | | | Agree | | | | | | | Disagree | | | | | | Strongly disagree | | | | | I don’t know | |
|  | How much do you disagree/agree with the following statements? SDF can be used without performing restorative treatment | Strongly agree | | | | | Agree | | | | | | | Disagree | | | | | | Strongly disagree | | | | | I don’t know | |
|  | Is SDF a good treatment for lesions that are in the aesthetic zone on primary teeth? | Yes | No | | | | | I don’t know | | | | | | | |  | | | | |  | | | | | |
|  | Is SDF a good treatment for lesions that are in the posterior zone on primary teeth? | Yes | No | | | | | I don’t know | | | | | | | |  | | | | |  | | | | | |
|  | Is SDF a good treatment for lesions that the aesthetic zone on permanent teeth? | Yes | No | | | | | I don’t know | | | | | | | |  | | | | |  | | | |  | |
|  | Is SDF a good treatment for lesions that are in the posterior zone on permanent teeth? | Yes | No | | | | | I don’t know | | | | | | |  | | |  | | | | |  | | | |
|  | Is SDF a good alternative treatment when patients take bisphosphonate medications? | Yes | No | | | | | I don’t know | | | | | | |  | | |  | | | | |  | | | |
|  | Is SDF a good alternative treatment when patients are undergoing /have recently undergone radiation therapy or chemotherapy? | Yes | No | | | | | I don’t know | | | | | | |  | | |  | | | | |  | | | |
|  | Is SDF a good alternative treatment for special needs patients? | Yes | No | | | | | I don’t know | | | | | | |  | | |  | | | | |  | | | |
|  | Is SDF a good alternative treatment when patients have severe dental anxiety? | Yes | No | | | | | I don’t know | | | | | | |  | | |  | | | | |  | | | |
|  | Is SDF a good alternative treatment for low-income patients? | Yes | No | | | | | I don’t know | | | | | | |  | | |  | | | | |  | | | |
|  | Is SDF a good alternative treatment for patients with behavioural issues? | Yes | No | | | | | I don’t know | | | | | | |  | | |  | | | | |  | | | |
| **Domain 4** | What are your doubts about SDF? Poor scientific evidence | Yes | No | | | | | I don’t know | | | | | | |  | | |  | | | | |  | | | |
|  | What are your doubts about SDF? Permanent discoloration of treated teeth | Yes | No | | | | | I don’t know | | | | | | |  | | |  | | | | |  | | | |
|  | What are your doubts about SDF? Cost for the patients | Yes | No | | | | | I don’t know | | | | | | |  | | |  | | | | |  | | | |
|  | What are your doubts about SDF? Failure to restore functional anatomy of teeth | Yes | No | | | | | I don’t know | | | | | | |  | | |  | | | | |  | | | |
|  | What are your doubts about SDF? Concern for patient satisfaction | Yes | No | | | | | I don’t know | | | | | | |  | | |  | | | | |  | | | |
|  | What are your doubts about SDF? Off-label use | Yes | No | | | | | I don’t know | | | | | | |  | | |  | | | | |  | | | |
|  | How often did you use SDF to treat tooth sensitivity? | Very often | | | | | | | Often | | | | | | | | Rarely | | | | | | | Never | | |
|  | How often did you use SDF to prevent dental caries? | Very often | | | | | | | Often | | | | | | | | Rarely | | | | | | | Never | | |
|  | How often did you use SDF to arrest dental caries in primary teeth? | Very often | | | | | | | Often | | | | | | | | Rarely | | | | | | | Never | | |
|  | How often did you use SDF to arrest dental caries in permanent teeth? | Very often | | | | | | | Often | | | | | | | | Rarely | | | | | | | Never | | |
|  | How often did you use SDF to definitively treat dental caries without restorative treatment? | Very often | | | | | | | Often | | | | | | | | Rarely | | | | | | | Never | | |
|  | Do you use SDF | Yes | | | | No | | | | | | | | | | |  | | | | | | |  | | |
|  | What kind of SDF’s protocol do you use? | One-shot | | Applications every week for 1 month | | | | | Application every 3 months | | | | Application every 6 months | | | | Application every 12 months | | | | | I am guided by the clinical examination and the number and frequency of lesions | | | | I don’t use SDF |
|  | Do you expect to increase your future usage of SDF? | Yes | | | | No | | | | | | |  | | | | | |  | | | | | | | |

Table S1: Demographic characteristics expressed as number of participants and percentage. (Participants 3337).

| **Domain 1: demographic characteristics** | | | | | | | | | | |
| --- | --- | --- | --- | --- | --- | --- | --- | --- | --- | --- |
| Item | N (%) | | | | | | | | | |
| **Age Range** | | <30 years | 31-40 years | | 41-50 years | 51-60 years | | > 60 years | |  |
|  |  | 322 (9.6) | 704 (21.1) | | 737 (22.1) | 776 (23.3) | | 798 (23.9) | |  |
| **Work experience** | | < 5 years | 5-10 years | | 11-20 years | > 20 years | | |  |  |
|  |  | 409 (12.3) | 438 (13.1) | | 793 (23.8) | 1697 (50.8) | | |  |  |
| **Practice/employment situation** | | Private clinic | | Public clinic | | | Both | | |  |
|  |  | 2900 (86.9) | | 160 (4.8) | | | 277 (8.3) | | |  |
| **Workplace** | | Large city | | Moderate/Small city | | | Rural | | |  |
|  |  | 1071 (32.1) | | 1595 (47.8) | | | 671 (20.1) | | |  |
| **Type of patients prevalently treated** | | Adults | | Children (<14 years) | | | Elderly (>65 years) | | |  |
|  | | 2756 (82.6) | | 466 (13.9) | | | 115 (3.4) | | |  |
| **Treating special needs patients** | | Yes | | No | | |  | | |  |
|  | | 1408 (42.2) | | 1929 (57.8) | | |  | | |  |

Table S2. Reported use of SDF (yes or no) by questionnaire items (Domains 3 and 4).

| **Domain 3: dentist’s attitudes toward SDF** | | | |
| --- | --- | --- | --- |
| Item | SDF | No SDF | p-Value |
| **How much do you disagree/agree with the following statements?** |  |  |  |
| SDF can be used to arrest non-cavitated lesions | N = 652 | N = 1888 |  |
| Agree | 560 (85.9) | 1549 (82.0) | 0.03 |
| Disagree | 92 (14.1) | 339 (18.0) |  |
| SDF can be used to arrest enamel cavitated lesion | N = 652 | N = 1855 |  |
| Agree | 563 (86.3) | 1492 (80.4) | <0.01 |
| Disagree | 89 (13.6) | 363 (19.6) |  |
| SDF can be used to arrest dentin cavitated lesion | N = 635 | N = 1781 |  |
| Agree | 397 (62.5) | 956 (53.7) | <0.01 |
| Disagree | 238 (37.5) | 825 (46.3) |  |
| SDF can be used to arrest cavitated root caries | N = 621 | N = 1722 |  |
| Agree | 352 (56.7) | 879 (51.1) | 0.02 |
| Disagree | 269 (43.3) | 843 (48.9) |  |
| Infected soft dentin must be removed prior to applying SDF | N = 637 | N = 1750 |  |
| Agree | 452 (71.0) | 952 (54.4) | <0.01 |
| Disagree | 185 (29.0) | 798 (45.6) |  |
| SDF can be used also without performing restorative treatment | N = 608 | N = 1654 |  |
| Agree | 412 (67.8) | 999 (60.4) | 0.02 |
| Disagree | 196 (32.2) | 655 (39.6) |  |
| **Is SDF a good treatment for lesions that…** |  |  |  |
| are in the aesthetic zone on primary teeth? | N = 606 | N = 1784 |  |
| Yes | 168 (27.7) | 217 (12.2) | <0.01 |
| No | 438 (72.3) | 1567 (87.8) |  |
| are in the posterior zone on primary teeth? | N = 606 | N = 1784 |  |
| Yes | 272 (44.9) | 1081 (60.6) | <0.01 |
| No | 334 (55.1) | 703 (39.4) |  |
| are in the aesthetic zone on permanent teeth? | N = 606 | N = 1784 |  |
| Yes | 132 (21.8) | 178 (10.0) | <0.01 |
| No | 474 (78.2) | 1606 (90.0) |  |
| are in the posterior zone on permanent teeth? | N = 606 | N = 1784 |  |
| Yes | 347 (57.3) | 1301 (72.9) | <0.01 |
| No | 259 (42.7) | 483 (27.1) |  |
| **Is SDF a good alternative treatment…** |  |  |  |
| for special needs patients? | N = 623 | N = 1857 |  |
| Yes | 351 (56.3) | 1166 (62.8) | <0.01 |
| No | 272 (43.7) | 691 (37.2) |  |
| when patients have severe dental anxiety? | N = 623 | N = 1857 |  |
| Yes | 323 (51.9) | 1086 (58.5) | <0.01 |
| No | 300 (48.1) | 771 (41.5) |  |
| for patients with behavioural issues? | N = 623 | N = 1857 |  |
| Yes | 436 (70.0) | 1576 (84.9) | <0.01 |
| No | 187 (30.0) | 281 (15.1) |  |
| when patients take bisphosphonate medications? | N = 623 | N = 1857 |  |
| Yes | 82 (13.2) | 260 (14.0) | 0.647 |
| No | 541 (86.8) | 1597 (86.0) |  |
| for patients during/shortly after radiotherapy or chemotherapy? | N = 623 | N = 1857 |  |
| Yes | 146 (23.4) | 410 (22.1) | 0.518 |
| No | 477 (76.6) | 1447 (77.9) |  |
| for low-income patients? | N = 623 | N = 1857 |  |
| Yes | 158 (25.4) | 630 (33.9) | <0.01 |
| No | 465 (74.6) | 1227 (66.1) |  |
| **Domain 4: dentists’ behavior on the use of SDF in clinical practice** | | | |
| Item | SDF | No SDF | p-Value |
| **What are your doubts about SDF?** |  |  |  |
| Poor evidence-based | N = 618 | N = 1976 |  |
| Yes | 60 (9.7) | 157 (8.0) | 0.194 |
| No | 558 (90.3) | 1819 (92.0) |  |
| Permanent discoloration of treated teeth | N = 618 | N = 1976 |  |
| Yes | 422 (68.3) | 1806 (91.4) | <0.01 |
| No | 196 (31.7) | 170 (8.6) |  |
| Cost for the patients | N = 618 | N = 1976 |  |
| Yes | 47 (7.6) | 87 (4.4) | <0.01 |
| No | 571 (92.4) | 1889 (95.6) |  |
| Failure to restore functional anatomy of teeth | N = 618 | N = 1976 |  |
| Yes | 220 (35.6) | 880 (44.5) | <0.01 |
| No | 398 (64.4) | 1096 (55.5) |  |
| Concern for patient satisfaction | N = 618 | N = 1976 |  |
| Yes | 192 (31.1) | 668 (33.8) | 0.225 |
| No | 426 (68.9) | 1308 (66.2) |  |
| Off-label use | N = 618 | N = 1976 |  |
| Yes | 96 (15.5) | 196 (9.9) | <0.01 |
| No | 522 (84.5) | 1780 (90.1) |  |
| **Do you expect to increase your future usage of SDF?** | N = 688 | N = 2649 |  |
| Yes | 560 (81.4) | 1901 (71.8) | <0.01 |
| No | 128 (18.6) | 748 (28.2) |  |
